# Magnetic Properties of Fibonacci-Modulated Fe-Au Multilayer Metamaterials

**DOI:** 10.3390/ma10101209

**Published:** 2017-10-20

**Authors:** Tomomi Suwa, Satoshi Tomita, Nobuyoshi Hosoito, Hisao Yanagi

**Affiliations:** Graduate School of Materials Science, Nara Institute of Science and Technology, Nara 630-0192, Japan; suwa.tomomi.sm4@ms.naist.jp (T.S.); hosoito@ms.naist.jp (N.H.); yanagi@ms.naist.jp (H.Y.)

**Keywords:** magnetic multilayers, one-dimensional metamaterials, Fibonacci sequence

## Abstract

Herein we experimentally study magnetic multilayer metamaterials with broken translational symmetry. Epitaxially-grown iron-gold (Fe-Au) multilayers modulated using Fibonacci sequence—referred to as magnetic inverse Fibonacci-modulated multilayers (IFMs)—are prepared using ultra-high-vacuum vapor deposition. Experimental results of in-situ reflection high-energy electron diffraction, magnetization curves, and ferromagnetic resonance demonstrate that the epitaxially-grown Fe-Au IFMs have quasi-isotropic magnetization, in contrast to the in-plane magnetization easy axis in the periodic multilayers.

## 1. Introduction

Multilayers are the most feasible structures for realizing one-dimensional (1D) metamaterials. For example, a class of photonic 1D metamaterials called hyperbolic metamaterials [1,2,3], which consist of metal-dielectric multilayers and realize a highly anisotropic medium with hyperbolic dispersion relations, has attracted much interest. By breaking translational symmetry (i.e., by introducing quasi-periodicity in the photonic hyperbolic metamaterials), enhancement of light–matter interaction was experimentally and theoretically demonstrated [4]. Quasi-periodic order modulates interaction between metallic layers in the metamaterials, bringing about the modification in local density of states. This modification leads to an enhancement of the decay rate of quantum emitters on the metamaterials. The enhancement arises from the localized lattice-like state inherent for self-similar quasi-periodic order, and such a localized state does not exist in periodically stratified metamaterials [5]. These results indicate that quasi-periodic 1D metamaterials are good model systems for modulating the coupling between constituents in 1D metamaterials.

In this communication, we experimentally study quasi-periodic magnetic 1D metamaterials, in other words, 1D metamaterials with simultaneous broken translational and time-reversal symmetries. In the metamaterials, the interactions between magnetic layers are modulated. Periodic magnetic multilayers (PMs) have been studied since the early 1990s [6]. In these studies, transition-metal PMs separated by noble-metal spacers have shown intriguing magnetic properties, such as perpendicular magnetic anisotropy [7,8]. In particular, much attention has been paid to iron-gold (Fe-Au) PMs, because these PMs have demonstrated not only perpendicular magnetization but also oscillatory coupling between ferromagnetic layers caused by the Ruderman-Kittel-Kasuya-Yosida interaction [9] and enhanced magneto-optical Kerr effects [10]. In contrast to the periodic counterpart, magnetic quasi-periodic multilayers remain experimentally unaddressed, although theoretical calculations have predicted anomalous magnetic resistance [11] and ferromagnetic resonance (FMR) [12].

Here we use the Fibonacci sequence frequently observed in nature to break the translational symmetry and induce modulation in the magnetic multilayers. Epitaxially-grown Fe-Au multilayers modulated using the Fibonacci sequence—herein referred to as Fe-Au inverse Fibonacci-modulated multilayers (IFMs)—are prepared using ultra-high-vacuum vapor deposition. The Fe-Au IFMs are studied using in-situ reflection high-energy electron diffraction (RHEED), magnetization, and FMR measurements. We reveal that IFMs have quasi-isotropic magnetic properties.

## 2. Experimental Procedures

**Figure 1:**
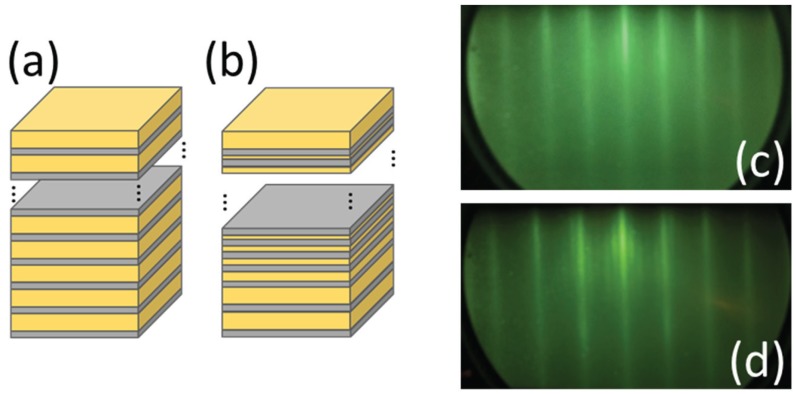
Schematic illustrations of Fe-Au (**a**) periodic multilayers (PMs) and (**b**) inverse Fibonacci-modulated multilayers (IFMs). The in-situ RHEED patterns of Au(100) plane (**c**) before and (**d**) after the deposition of Fe-Au IFMs.

Figure 1a,b show schematic illustrations of the sample structures. We prepared two types of multilayers: Fe-Au PMs (Figure 1a) and Fe-Au IFMs (Figure 1b). Gray and yellow colors in Figure 1a,b correspond to Fe and Au layers, respectively. Fe and Au layers are known to have a very small mismatch of lattice spacing [13,14]. The Fe-Au multilayers were epitaxially grown on single-crystal MgO (100) substrates using ultra-high-vacuum vapor deposition equipped with an in-situ RHEED apparatus (EV-10S, Eiko, Tokyo, Japan). The base pressure was lower than 2 × $10^{-9}$ Torr, and the deposition rate was 0.1 Å per second. In this paper, the number next to the element symbols corresponds to film thickness in the unit of Å. In order to obtain epitaxially-grown multilayers, a seed Fe10 layer followed by a buffer Au200 layer was deposited on the MgO substrates at a substrate temperature of 200 ${}^{\circ }$C. The epitaxial growth of (100) planes of Fe and Au on the substrate was confirmed by in-situ RHEED. Figure 1c shows in-situ RHEED patterns of the Au(100) plane after the buffer layer deposition. Streak lines in the RHEED patterns demonstrate the Au buffer layer epitaxially grown on the Fe seed layer.

The Fe layer thickness was identical (5 Å) in both multilayers. The Fe-Au PMs sample consisted of Fe5 and Au30 layers alternately stratified thirteen times on the buffer layer. The PMs sample is thus represented by MgO/seed Fe10/buffer Au200/[Fe5/Au30]_13_. In contrast, the Au layer thickness in the Fe-Au IFMs is not constant, but gradually decreased following the Fibonacci number, which can be written by a recursive formula given as

(1) $$F_{n+2}=F_{n}+F_{n+1},$$

where $F_{0}=0$ and $F_{1}=1$. In this way, the *n*th Au layer has a thickness corresponding to 30 Å divided by $F_{n}$ up to n=6. The IFMs were repeated twice in the sample, and finally terminated by Fe5 and Au30; namely, the IFMs sample is represented by MgO/seed Fe10/buffer Au200/[Fe5/Au30/Fe5/Au30/Fe5/Au15/Fe5/Au10/Fe5/Au6/Fe5/Au4]_2_/Fe5/Au30. Figure 1d shows in-situ RHEED patterns after deposition of Fe-Au IFMs. The RHEED pattern is very similar to that before the multilayer deposition, as shown in Figure 1c. Streak lines in Figure 1d demonstrate the epitaxial growth of the final capping layer, indicating that the multilayer was epitaxially grown on the buffer layer. Moreover, satellite lines between the streak lines indicate the presence of pure Au surface and no intermixing between Fe and Au in the multilayer. Although not shown here, the control Fe-Au PMs sample shows similar streak lines in the in-situ RHEED after the deposition.

**Figure 2:**
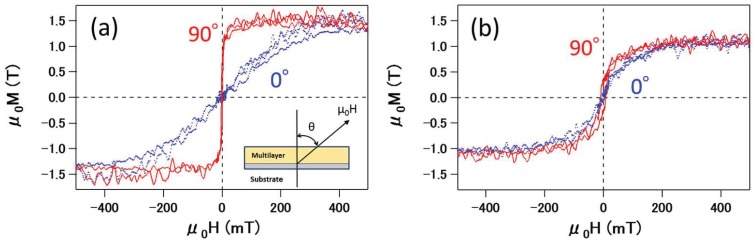
Magnetic hysteresis loops of (**a**) Fe-Au PMs and (**b**) Fe-Au IFMs samples. The $\mu_{0}H$ in the magnetization measurements was applied in the perpendicular direction (θ = 0${}^{\circ }$) or parallel direction (θ = 90${}^{\circ }$) to the film surface.

We measured the magnetic hysteresis loops of the multilayer samples using a vibrating sample magnetometer (VSM; BHV-525RSCM, Riken Denshi, Tokyo, Japan). The VSM measurements were carried out at room temperature. The dc magnetic fields $\mu_{0}H$ up to 500 mT were generated using an electromagnet. The $\mu_{0}H$ configuration is illustrated in the inset of Figure 2a. The $\mu_{0}H$ in the magnetization measurements was applied in the perpendicular direction (θ = 0${}^{\circ }$) or parallel direction (θ = 90${}^{\circ }$) to the film surface. The angle-resolved FMR of the multilayer samples was investigated using an electron spin resonance spectrometer (JES-FA100N, JEOL, Tokyo, Japan) equipped with a TE_011_ cavity for the *X*-band (9.8 GHz) microwave. The FMR measurements were conducted at room temperature. The FMR spectra were measured at every 15${}^{\circ }$, while the sample was rotated between θ = 0 and 180${}^{\circ }$. The spectra between 45${}^{\circ }$ and 135${}^{\circ }$ were obtained in a magnetic field range between 100 and 500 mT, whereas the other spectra were obtained in a magnetic field range between 150 and 650 mT.

## 3. Results and Discussion

Figure 2 shows the magnetic hysteresis loops of Fe-Au (a) PMs and (b) IFMs samples. The red solid and blue dotted curves correspond to the hysteresis loops at θ = 90${}^{\circ }$ and θ = 0${}^{\circ }$, respectively. The magnetization is normalized by the total Fe volume in multilayers, including the seed Fe layer.

Figure 2a shows the magnetization curves of Fe-Au PMs. The in-plane magnetization at θ = 90${}^{\circ }$ was saturated at approximately 100 mT, whereas the out-of-plane magnetization at θ = 0${}^{\circ }$ was saturated at approximately 300 mT. Figure 2a shows that the saturation magnetization $\mu_{0}M_{s}$ was about 1.51 T, which is 30% smaller than that of the bulk Fe saturation magnetization value (2.16 T) [15]. The decrease in saturation magnetization of the multilayer is caused by a decrease in Curie temperature for constituent 5 Å Fe layers. In Figure 2a, magnetization at θ = 0${}^{\circ }$ was saturated by applying a magnetic field of approximately 300 mT, which is much smaller than the demagnetization field of approximately 2.2 T [15]. This magnetization curve at θ = 0${}^{\circ }$ indicates that the PMs sample has out-of-plane magnetic anisotropy as well as in-plane anisotropy. However, in the PMs sample, the in-plane magnetic anisotropy is larger than the out-of-plane anisotropy. Since the anisotropy energy $|E_{A}|$ is calculated by

(2) $$|E_{A}|=\left|\int H_{∥}dM-\int H_{⊥}dM\right|,$$

the anisotropy energy for the PMs sample $|E_{A}^{\mathrm{PM}}|$ is 2.4 × 10${}^{4}$ J/m^3^. In this way, the in-plane magnetization is dominant in the PMs sample.

Figure 2b shows the magnetization curves of the Fe-Au IFMs sample. In sharp contrast to the PMs sample, the magnetization curves of the IFMs sample both at θ = 0 (blue) and 90${}^{\circ }$ (red) are similar. The magnetization was saturated by applying a magnetic field of approximately 300 mT. The saturation magnetization of the IFMs sample was about 1.08 T, which is 19% smaller than that of the PMs sample, even though the total Fe volume in these two samples was identical. A smaller saturation magnetization of the IFMs sample compared to the PMs sample is traced back to the interface between Fe and very thin Au layers. It is known that a very thin Au layer is initially grown like islands on the Fe layer, resulting in rough interfaces between Fe and Au layers. Because the IFMs sample contains very thin Au layers, the roughness at the interfaces is enhanced. In this way, the rough Fe layer in the IFMs sample results in a smaller saturation magnetic field . The intermixing of Fe and Au at the interface could be an alternative origin of the decrease in the saturation magnetization [16,17], while the streak lines in the RHEED patterns in Figure 1d indicate no intermixing between Fe and Au in the multilayer.

The IFMs magnetization curves in Figure 2b indicate that $|E_{A}^{IFM}|$ is 6.4 × 10${}^{2}$ J/m^3^. When $|E_{A}^{IFM}|$ is compared to $|E_{A}^{PM}|$, the magnetization process in the IFMs sample is quasi-isotropic. The isotropic magnetization process is most likely caused by the cancellation of shape anisotropy due to the demagnetization field by perpendicular anisotropy due to the interface effects at θ = 0${}^{\circ }$. The effective anisotropy magnetic field $H_{\mathrm{keff}}$ is represented by

(3) $$|H_{\mathrm{keff}}|=\left|\left(\frac{K_{u}}{2}\right)-M\right|,$$

where $K_{u}$ and *M* are the anisotropy constant and magnetization, respectively. The magnitude of perpendicular magnetization of the IFMs sample is evaluated to be approximately 2.2 T.

**Figure 3:**
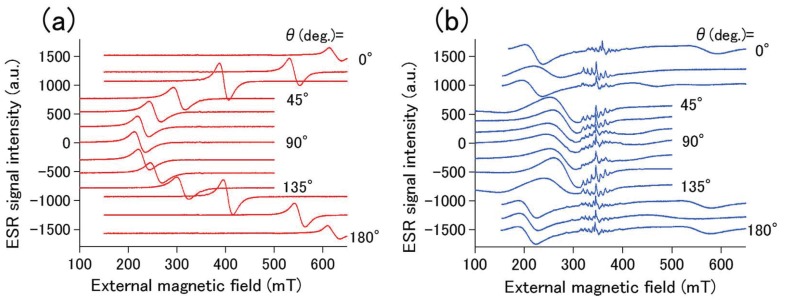
Angle-resolved ferromagnetic resonance (FMR) spectra of (**a**) Fe-Au PMs and (**b**) Fe-Au IFMs samples.

Figure 3 shows the angle-resolved FMR spectra of Fe-Au (a) PMs and (b) IFMs samples. Angle-resolved FMR spectra of the Fe-Au PMs sample in Figure 3a show that, with θ = 0${}^{\circ }$, an FMR signal is observed at approximately 600 mT. As θ is increased to 90${}^{\circ }$, the FMR signal shifts to a lower magnetic field. The resonance signal arrives at approximately 250 mT when θ = 90${}^{\circ }$. With a further increase in θ up to 180${}^{\circ }$, the signal shifts back to a higher magnetic field. This signal shift is attributed to the Kittle mode FMR, corresponding to the uniform precession of electron spins in Fe layers. The shift of the FMR signal is caused by the magnetic shape anisotropy in the PMs sample, because the shift direction is consistent with large in-plane magnetization revealed by magnetization measurements in Figure 2a.

Figure 3b shows the angle-resolved FMR of the Fe-Au IFMs sample. Note that small signals around 350 mT in Figure 3b are attributed to the MgO(100) substrate. The intensity of FMR signals in Figure 3b is much smaller than that in Figure 3a. An FMR signal is observed at approximately 200 mT with θ = 0${}^{\circ }$. While this signal is slightly shifted to a higher magnetic field with an increase in θ up to 90${}^{\circ }$, the shift variation is small. These FMR results in Figure 3b are consistent with the magnetization measurement results demonstrating the isotropic magnetization process observed in Figure 2b.

**Figure 4:**
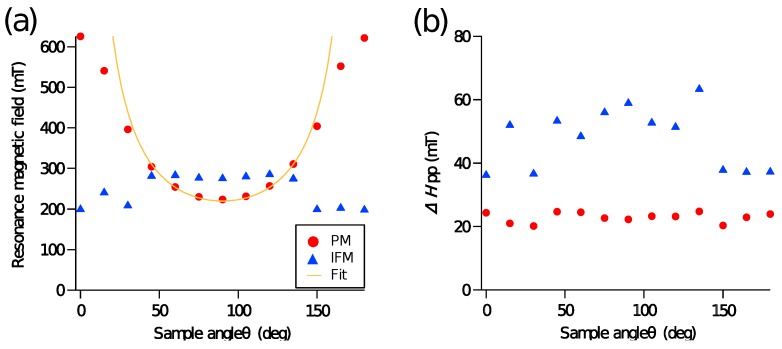
(**a**) Angle dependence of resonance magnetic field $H_{0}$ and (**b**) angle dependence of peak-to-peak linewidth Δ*H*_pp_. The PMs and IFMs samples correspond, respectively, to red circles and blue triangles. The yellow line represents a fitting curve.

Resonance magnetic field $H_{0}$ and peak-to-peak linewidth $\Delta H_{\mathrm{pp}}$ are plotted as a function of magnetic field angle θ in Figure 4a,b, respectively. The PMs and IFMs samples correspond respectively to red circles and blue triangles. In Figure 4a, $H_{0}$ of the IFMs sample is fitted using the Kittel’s equation as

(4) $${\left(\frac{\omega }{\gamma }\right)}^{2}=\left(H\frac{\sin\beta }{\sin\theta }\right)\left[\left(\frac{2K_{u}}{M}\right)-M\right]\cos\left(2\theta \right),$$

where ω, γ, β, and θ are the angular frequency, the gyromagnetic constant, the angle of external magnetic field, and the angle of magnetization, respectively. The yellow line in Figure 4a represents a fitting curve. The fitting curve does not reproduce the resonance fields experimentally observed in the regions below 30${}^{\circ }$ and above 150${}^{\circ }$ because the Kittel’s equation in Equation (4) is applicable for thin films with in-plane uniaxial magnetic anisotropy. These results indicate that the PMs sample has perpendicular magnetic anisotropy induced by the magnetocrystalline anisotropy at the Fe-Au interfaces. Figure 4b shows that $\Delta H_{\mathrm{pp}}$ is not dependent on the magnetic field angle in both the PMs and IFMs samples. The $\Delta H_{\mathrm{pp}}$ of the IFMs sample is approximately twice of that of the PMs sample. This large $\Delta H_{\mathrm{pp}}$ indicates that the Gilbert damping of the IFMs sample is larger than that of the PMs sample. Additionally, the large distribution of local demagnetization field (i.e., magnetic dipole field) and magnon scattering [18,19] due to the roughness between Fe and Au layers are another possible origins for the large $\Delta H_{\mathrm{pp}}$ in the IFMs sample.

Several studies on Fe-Au thickness-modulated multilayers [20,21] have been reported so far. Almost all of the previous multilayers have in-plane magnetization. In contrast, the present IFMs sample shows perpendicular anisotropy competing with the shape anisotropy. On the top of the IFMs sample with very thin Au layers, an atomic layer superlattice (for example, an L1${}_{0}$ type superlattice) could show magnetization in a direction perpendicular to the film surface [22,23]. Whereas the true origin of the perpendicular anisotropy is still unclear, it is reasonable to think that interfaces between Fe and Au layers play an important role [24,25,26]. Additionally, although not shown here, FMR spectra and magnetization curves very similar to those of the PMs sample were observed for a sample with a similar but slightly changed structure from the IFMs sample. These results indicate that a small variation in the multilayer sequence and spacer thickness causes a significant change in the magnetic properties of the IFMs sample. Therefore, we conclude that the perpendicular anisotropy is traced back to the IFMs structure with quasi-periodicity.

## 4. Conclusions

Ferromagnetic resonance in Fibonacci-modulated magnetic metamaterials was investigated. We prepared Fe-Au PMs and IFMs samples using ultra-high-vacuum vapor deposition. The in-situ RHEED highlighted epitaxial growth of Fe and Au layers in both the PMs and IFMs samples. The VSM and FMR demonstrated that the IFMs sample had a quasi-isotropic magnetization process, while the PMs sample had in-plane magnetization. The isotropic magnetization process is traced back to the cancellation between shape anisotropy due to the demagnetization field and perpendicular anisotropy due to the interface effects. This study paves the way to the novel magnetic metamaterials with quasi-periodicity.
